# Certain Provincial Hospitals: Their Dissolution or Reform

**Published:** 1914-07-25

**Authors:** 


### The Hospital

The Workers' Newspaper of Administrative Medicine and Institutional Life, Administration, National Insurance and Health.

No. 1466, Vol. LVI. SATURDAY, JULY 25, 1914. PRICE ONE PENNY.

### CERTAIN PROVINCIAL HOSPITALS: THEIR DISSOLUTION OR REFORM.

The time has come when it is essential to call attention to a few of the least efficient and most sleepy general hospitals throughout the country, where things have got largely into a rut, and where some of the worst features of the old system of patronage may prevail. Fortunately, there are less than a dozen of these institutions to be found, in England at any rate, and the chief of them are to be met with in some of the southern counties. In these institutions it sometimes happens, as at Portsmouth, that, owing to the control having gradually been centred in a portion only of the population, the hospital is not generally popular, it is sadly in need of greater financial support, and it lacks, from the administrative and managerial point of view, initiative and knowledge. We may say quite frankly that such an absence of initiative and knowledge demands the services of a trained and capable man as chief of the administrative staff, who should be adequately paid and endowed with full authority. Such a competent official is essential to the successful administration of a modern hospital everywhere in the present day. Some of the people of Portsmouth declare that they lack this essential authority in their hospital system, and that they further have to suffer from the evils attending cliqueism in the management.

We have called attention to the condition of the Portsmouth Hospital more than once before. The causes of most of the trouble still exist, we understand, and have been aggravated by time and the failure to keep the various departments of the hospital up to date. It would be an immense advantage to the cause of efficiency at this hospital, and should move the hearts and consciences of the whole of the inhabitants of Portsmouth, if the medical officer of health for the district would promptly make an inspection of the pathological department, including the mortuary and its appurtenances, write an exact account of what he finds and sees, and publish it in the Portsmouth papers. Anyone who doubts the effect of such an action or its justification should make it his business, whether he has technical knowledge or not, to inspect these portions of the Royal Portsmouth Hospital, and to be sure that he sees the whole of them.

Another justifiable reason for the non-provision of an adequate income for this hospital would seem to be the existence of that old and archaic tyranny an Admission Committee, before whom each patient seeking treatment has to appear if he or she requires admission or treatment. The Governors' complaint is that they do not get adequate support from the working and other classes who benefit by the treatment the patients obtain when they manage to enter the narrow portal of admission which is so carefully guarded by the Admission Committee. We wonder if the members of this committee have ever thought what the effect of their system would be upon themselves or members of their families if they had to come some miles, to give up a day's work to attend before this committee, and to be cross-examined upon a number of points of an intimate personal nature, including the condition of their family and other private matters? Then in the result to be told that their names will, or will not, be entered on the waiting list, and that they will be invited to come again when a vacant bed is available.

For nearly fifty years we have been fighting to free the voluntary hospitals from the curse of the Admission Committee system and all it involved. It was with amazement we learnt, quite accidentally, that this system still exists in full force and action in the town of Portsmouth, where the voluntary hospital is administratively notoriously behind the times, and not unnaturally languishing for want of adequate support. Unless the committee of management exhibit the intelligence and energy required to abolish the Admission Committee forthwith, they do not deserve and cannot expect to obtain more, but must be prepared to receive continuously less money from the public year by year. That will be one necessary consequence of continuing an evil system which every well-administered hospital in the land has shed for decades. Moreover, the want of administrative acumen at Portsmouth is strikingly proved by the fact that the discredited system of "an Admission Committee " has been deprecated for years by members of the medical staff, to their great honour and credit, as well as by the patients who required hospital care. The only basis of argument, so far as we have been able to ascertain, which the supporters of this abomination cling to is the contention that the Admission Committee is often able to wring contributions from the unfortunate patients which would otherwise not be received by the hospital. This is a financial point, and a reference to the accounts reveals its hollowness. The total amount received from patients' payments in the year 1912 (the last year for which the audited figures are available) was a miserable £382. The number of in-patients treated to a conclusion was 2,043. Having regard to the time expended by the Admission Committee each week in their arduous duties, an outside sum of 3s. per patient cannot be regarded by the most modest member of that committee as adequate testimony to the value of his or her eminent services. We wish to make it quite clear that we have no doubt whatever that the members of the committee are conscientious in endeavouring to do their duty according to their lights. It is with a view to arousing them to an adequate sense of the proportion of things that we have ventured to take the Admission Committee as an illustration of the causes which collectively underlie the lamentable failure of the honourable efforts in the cause of the Portsmouth Hospital.

